# Lovastatin for adult patients with dengue: protocol for a randomised controlled trial

**DOI:** 10.1186/1745-6215-13-203

**Published:** 2012-10-31

**Authors:** James Whitehorn, Nguyen Van Vinh Chau, Nguyen Thanh Truong, Luong Thi Hue Tai, Nguyen Van Hao, Tran Tinh Hien, Marcel Wolbers, Laura Merson, Nguyen Thi Phuong Dung, Rosanna Peeling, Cameron Simmons, Bridget Wills, Jeremy Farrar

**Affiliations:** 1 Department of Clinical Research, London School of Hygiene and Tropical Medicine, London, UK; 2 Hospital for Tropical Diseases Oxford University Clinical Research Unit, Wellcome Trust Major Overseas Programme, Ho Chi Minh City, Vietnam; 3 Hospital for Tropical Diseases, Ho Chi Minh City, Vietnam; 4 Centre for Tropical Medicine, University of Oxford, Oxford, UK

**Keywords:** Clinical trial, Dengue, Lovastatin, Statins

## Abstract

**Background:**

Dengue is the most important vector-borne viral infection of man, with approximately 2 billion people living in areas at risk. Infection results in a range of manifestations from asymptomatic infection through to life-threatening shock and haemorrhage. One of the hallmarks of severe dengue is vascular endothelial disruption. There is currently no specific therapy and clinical management is limited to supportive care. Statins are a class of drug initially developed for lipid lowering. There has been considerable recent interest in their effects beyond lipid lowering. These include anti-inflammatory effects at the endothelium. In addition, it is possible that lovastatin may have an anti-viral effect against dengue. Observational data suggest that the use of statins may improve outcomes for such conditions as sepsis and pneumonia. This paper describes the protocol for a randomised controlled trial investigating a short course of lovastatin therapy in adult patients with dengue.

**Methods/design:**

A randomised, double-blind, placebo-controlled trial will investigate the effects of lovastatin therapy in the treatment of dengue. The trial will be conducted in two phases with an escalation of dose between phases if an interim safety review is satisfactory. This is an exploratory study focusing on safety and there are no data on which to base a sample size calculation. A target sample size of 300 patients in the second phase, enrolled over two dengue seasons, was chosen based on clinical judgement and feasibility considerations. In a previous randomised trial in dengue, about 10% and 30% of patients experienced at least one serious adverse event or adverse event, respectively. With 300 patients, we will have 80% power to detect an increase of 12% (from 10% to 22%) or 16% (from 30% to 46%) in the frequency of adverse events. Furthermore, this sample size ensures some power to explore the efficacy of statins.

**Discussion:**

The development of a dengue therapeutic that can attenuate disease would be an enormous advance in global health. The favourable effects of statins on the endothelium, their good safety profile and their low cost make lovastatin an attractive therapeutic candidate.

**Trial registration:**

International Standard Randomised Controlled Trial Number ISRCTN03147572

## Background

Dengue is the most common and important vector-borne viral infection of man, with at least 2 billion people living in areas of risk [1]. Clinical dengue varies from asymptomatic infection to severe disease characterised by shock and haemorrhage. There are currently no specific therapeutic agents and disease management is limited to careful fluid management [2,3].

### Statins

Statins are inhibitors of 3-hydroxy-3-methylglutaryl-coenzyme A (HMG-CoA) reductase. They were first used clinically in the late 1980s and quickly became established as an effective drug for both lipid lowering and mortality reduction in cardiovascular disease [4]. They are currently one of the most prescribed classes of drugs globally. Statins have an excellent safety profile [5]. The most common adverse effects are rises in the level of liver transaminases and myopathy. These effects are rare and appear to be dose-related [5,6]. More recent research has demonstrated that statins have additional beneficial effects. These pleiotropic effects include the restoration or improvement of endothelial cell function, an increased production of nitric oxide, and a reduction in the release of cytokines and acute phase proteins. These effects lead to a reduction of inflammation within the vessel wall [7,8]. Observational studies suggest that statin therapy may result in improved outcomes for a diverse range of conditions, including sepsis and pneumonia [9-13]. In addition, there are a number of on-going randomised clinical trials to assess the therapeutic efficacy of statins in sepsis and acute lung injury (NCT00528580, NCT00676897, NCT00979121).

### Statins and dengue

One of the major features of both dengue and sepsis is widespread vascular endothelial disruption resulting, in part, from exposure to inflammatory mediators [14-16]. This suggests that in view of their pleiotropic effects, it is plausible that statins may favourably augment the pathophysiological mechanisms of these two conditions. A study in healthy volunteers showed that the endotoxin-induced inflammatory response was suppressed in those receiving a statin at a high dose (simvastatin 80 mg), perhaps explaining the beneficial effect observed in sepsis [8,17]. Furthermore, *in vitro* work has demonstrated that lovastatin may have an anti-viral effect in dengue by reducing virion assembly [18,19].

### Choice of study drug

We have chosen lovastatin as the study drug for both scientific and pragmatic reasons. In light of the US Food and Drug Administration’s safety recommendations about the risks of muscle toxicity with simvastatin, there were concerns from the regulatory authorities in Vietnam about the safety of using this agent in dengue. An additional reason for our choice was the dengue anti-viral effect observed with lovastatin in *in vitro* experiments [18]. As this effect may also be observed *in vitro*, we believe it is rational to test the drug used in the original work. Furthermore, lovastatin is commonly prescribed and available in Vietnam as a generic and will therefore be immediately available to patients if there is a positive result from the trial.

### Aims of the trial

Typically, severe vascular leakage and shock occur around the fifth day of illness in patients with dengue [20]. It is possible that initiation of statin therapy early in the course of the illness may prevent or favourably modulate these effects. As the proportion of patients who develop shock or other complications after presentation to hospital is low (approximately 5%), a large trial would be required to demonstrate a clinical benefit [2,21]. However, this study presents an opportunity to assess formally the safety of using statins in the treatment of this disease. In addition, such a study will also provide an opportunity to investigate the effect of statin usage on the immune response during dengue infection and to generate preliminary data for planning a Phase III trial in the future.

Although there is extensive experience of using statins as a lipid-lowering agent, and an increase in the amount of observational data from their use in critically ill patients, this will be the first study looking at the use of statins in dengue. We propose to investigate the effect of lovastatin for 5 days in adult dengue patients presenting in the first 72 hours of illness. The rational of a 5-day treatment course is to cover the transient ‘critical’ phase of infection when complications arise, which typically occurs around the fifth day of illness [3]. Our proposed treatment course will start on the second or third day of illness and continue to the seventh or eighth day of illness, which is into the recovery phase. As this is the first study investigating statin therapy in dengue with a particular focus on safety, we propose a dose-escalation study, investigating 40 mg lovastatin versus placebo with a safety review after recruitment of the first 30 patients. If this review is satisfactory, we will increase the lovastatin dose to 80 mg and conduct a further safety review after the next 30 patients.

## Methods/design

### Design

This study is a randomised, placebo-controlled, double-blind trial investigating lovastatin therapy in Vietnamese adults with dengue infection. The trial will be conducted in two phases, with an escalation of dose between phases if the results of an interim data review show no safety concerns within the first cohort of patients treated with the lower dose.

Patients will be followed for clinical and laboratory endpoints in hospital until study day 6 (or daily as out-patients from discharge to day 6) and reviewed at an outpatient visit on day 28.

### Inclusion and exclusion criteria

All patients aged 18 or more presenting to the Hospital of Tropical Diseases, Ho Chi Minh City with a clinical suspicion of dengue, less than 72 hours of fever and a positive rapid test for dengue non-structural protein 1 (NS1) will be eligible for recruitment into the study. Exclusion criteria are: signs or symptoms suggestive of another acute infectious disease, alanine transaminase levels greater than 150 U/l, creatine kinase levels greater than 1000 U/l, liver cirrhosis, myopathy, current or use within past week of statins, pregnancy and lactation. In addition, patients taking medications contraindicated for use with statins, for example, isoniazid for treatment of tuberculosis, will be excluded.

### Primary endpoint

The primary endpoint of this study is an evaluation of the safety and tolerability of lovastatin therapy in adult patients with dengue. Comparing the rates of adverse events between randomised treatment arms will assess this.

### Secondary endpoints

The secondary endpoints of this study are fever clearance time (see definition), plasma viraemia (area under the log-transformed viraemia curve from enrolment until study day 6), platelet nadir between day 3 and 8 of illness, maximum haematocrit between day 3 and 8 of illness, percentage increase in haematocrit between day 3 and 8 of illness from baseline, maximum alanine transaminase (ALT) and creatine kinase (CK) recorded between day 3 and 8 of illness, lowest oxygen saturation recorded between day 3 and 8 of illness, number of patients in each group requiring colloid, and disease progression as defined by one or more of the following: (a) admission to the ICU, (b) diagnosis of shock (see definition), (c) diagnosis of severe bleeding (see definition), (d) development of encephalitis, (e) death. In addition, quality of life scores obtained using a visual analogue scale will be compared between treatment groups. The times from enrolment to the first sample with viraemia less than 1000 copies/ml and the first negative NS1 antigenaemia sample will be compared between treatment groups.

### Definitions

1. Fever clearance time is the time from enrolment to the first time the temperature falls to <37.5°C and remains below this level for 48 hours.

2. Shock: cardiovascular decompensation requiring fluid resuscitation and considered to be due to plasma leakage.

3. Severe bleeding is clinically severe if it results in haemodynamic instability or requires fluid resuscitation or a blood transfusion. Any bleeding resulting in death and any intracranial bleed are considered severe.

4. Baseline haematocrit: the haematocrit value obtained at the day 28 follow-up visit, or (if the day 28 value is missing) the expected age- and sex-matched population value.

### Randomization procedure

Randomization to either treatment arm will be in a 1:1 ratio. Randomization will be stratified according to the ward of recruitment. A randomization list will be prepared and maintained confidentially from study staff by the clinical trials pharmacist. Block randomization using variable block sizes will be used.

A chronological log of all enrolled patients will be maintained. Each enrolled patient will be assigned the next available sequential study code. The assigned number will correspond to a coded, sealed, pre-packaged bottle containing six doses of either lovastatin or visually matched placebo. Blinding will be maintained amongst the attending physicians and nurses by ensuring that the study drug and the placebo have an identical appearance. In addition the administration schedule will be identical.

### Enrolment

Patients presenting to the out-patients department or in-patient wards with a clinical suspicion of dengue and less than 72 hours of fever will be identified to study staff. Study staff will approach these patients, check eligibility criteria and confirm dengue by NS1 rapid test. Eligible and consenting patients will have screening blood tests sent and provided these are satisfactory will be allotted the next consecutive study number and enrolled to the study.

### Safety reviews

This trial will be conducted in two phases with an escalation of dose if the results of an interim data review show no safety concerns within the first cohort treated with the lower dose. The dose will begin at 40 mg per day in cohort 1 and may continue at 80 mg per day in cohort 2. A DSMB (data and safety monitoring board) review will take place when day 6 study data are available from the 30th patient enrolled in cohort 1. If this review is satisfactory, the dose will be increased to 80 mg per day and recruitment into cohort 2 will commence. Further DSMB reviews will take place when day 6 study data are available from the 30th and 100th patients enrolled in cohort 2.

### Treatment and drug dispensation

Patients will be assigned to one of two treatment arms:

Active medicinal product: 40 mg (stage 1) or 80 mg (stage 2) lovastatin once daily for 5 days.

Placebo: visually matched placebo once daily for 5 days.

The first dose will be given as soon as practically possible after enrolment.

An unblinded study pharmacist will prepare study drug bottles centrally and will distribute the bottles as required. Drugs will be stored in accordance with the manufacturers’ recommendations in a secure area. Lovastatin and the placebo must be maintained below 25°C. All movements of study medication will be recorded. Both individual subject and overall drug accountability records will be kept up to date by the study staff.

### Data collection

#### Clinical evaluation

Patients will be followed by a study physician daily until discharge, and all signs and symptoms recorded in the case report form. An ultrasound scan will be performed on day 6 of illness to detect signs of plasma leakage. Clinical management decisions will remain in the hands of the attending ward doctors. In the event that shock or any other serious complication develops, the patient will be transferred to the appropriate ICU. Details of all adverse events will be recorded on specific forms, together with an assessment as to whether the event is likely to be related to any treatment received, and all serious events will be reported promptly to the DSMB.

Quality of life will be measured by questionnaire and visual analogue scale daily.

Patients who are fit to discharge on or after study day 3 may be followed as an outpatient until study day 6. All patients will be asked for attend a follow-up visit for review after 4 weeks.

#### Laboratory evaluation

Haematocrit, platelet and total cholesterol measurements will be carried out daily or more frequently if clinically indicated. These tests will be repeated at the follow-up visit.

Renal and liver function tests, electrolytes and coagulation profiles, will be carried out at enrolment, 48 hours later, day 5 or 6 of illness and at the follow-up visit. If the ALT measured 48 hours after enrolment is greater than 250 U/l, the study drug will be discontinued. It should, however, be noted that hepatic dysfunction might be secondary to dengue infection and could be positively affected by statin therapy.

Conventional serological and virological tests will be used to confirm dengue infection and identify the infecting serotype. Plasma samples collected at daily intervals until discharge (and daily until day 6 if discharged before day 6) will be assessed for viraemia levels, NS1 levels, and concentrations of various pro- and anti-inflammatory cytokines (TNF-α, IFN-γ, IL-6, IL-10).

DNA will be extracted from residual blood samples and genotyped for genetic variants known to be associated with severe dengue, for example, *MICB* and *PLCE1*[22].

### Statistical considerations

#### Sample size

This is an exploratory study focusing primarily on safety and there are no preliminary data regarding the effects of statins in dengue on which to base a sample size calculation. A target sample size of 300 patients in cohort 2, enrolled over two dengue seasons, was chosen based on clinical judgement and feasibility considerations. In a previous randomised trial in dengue, about 10% and 30% of patients experienced at least one serious adverse event or adverse event, respectively [23]. With 300 patients, we will have 80% power to detect an increase of 12% (from 10% to 22%) or 16% (from 30% to 46%) in the frequency of adverse events. In addition, this sample size ensures some power to explore the efficacy of statins. Specifically, this study will have 80% power to detect an increase in the rate of fever clearance by 40% due to statins. Based on simulations, we previously found that 30 patients give approximately 80% power to detect a 0.5 log10-copies/ml per day higher viraemia clearance, a reasonable estimate of what an effective anti-viral might achieve [24]. Thus, with 300 patients (a ten-fold higher sample size), we expect to be able to detect a (hypothetical) 0.16 log10-copies/ml per day higher viraemia clearance due to statins.

#### Statistical analysis

The primary analysis population will include all patients randomised to placebo from cohort 1 and all patients (regardless of treatment assignment) from cohort 2 according to the intention-to-treat principle. Owing to their low number, patients randomised to low-dose statins from cohort 1 will only be descriptively analysed.

The proportion of patients with any adverse events, any serious adverse events, or specific adverse events will be summarised and compared between the treatment arms based on Fisher’s exact test.

Pre-defined secondary endpoints will be compared between the two treatment arms based on linear regression for continuous endpoints, logistic regression for binary endpoints, and Cox regression for time-to-event endpoints. For laboratory markers, comparisons will be adjusted for the pre-dose value of the respective marker and the day of illness at enrolment; plasma viraemia and NS1-endpoints will additionally be adjusted for dengue serotype.

The clinical, virological and immunological findings will also be correlated with *MICB* and *PLCE1* genotype using descriptive statistical methods.

A detailed statistical analysis plan will be finalised prior to unblinding the study data base.

### Ethical considerations

#### Ethical approval

This protocol and both the patient information sheet and the consent form have been reviewed and approved by the Institutional Review Board of the Hospital for Tropical Diseases in Ho Chi Minh City, the Oxford Tropical Research Ethics Committee and the Ethics Committee of the London School of Hygiene and Tropical Medicine.

#### Informed consent and information sheet

All patients entering the study must give informed consent.

#### Withdrawal from the trial

Each participant has the right to withdraw from the study at any time. The reason for withdrawal will be recorded in the case report form.

#### Confidentiality

Patients who enter the trial will be given a unique identification number. This number will be used on both laboratory specimens and case report forms. The study wards and the research unit have the facilities to store study information securely.

#### The role of the data and safety monitoring board (DSMB)

An independent DSMB will be set up consisting of a biostatistician and senior clinical researchers with expertise in dengue and clinical trials. The DSMB will review the protocol and agree to a data review schedule and reporting requirements before the study commences. All data reviewed by the DSMB will be in the strictest confidence. A DSMB charter will outline its responsibilities and operation.

The DSMB will perform a safety review after day 6 data are available for the first 30 patients enrolled (Cohort 1: 40 mg lovastatin daily). This review will be based on a report created by the DSMB statistician containing unblinded summary tables of baseline demographics, serious adverse effects, adverse effects and disease progressions, as well as viraemia curves. If no safety concerns are identified, the lovastatin dose will be increased to 80 mg daily and recruitment will commence in cohort 2. Enrolment will continue in cohort 1 while awaiting the outcome of the DSMB review. Additional safety reviews will take place after the day 6 data are available for the 30th and 100th patients in cohort 2.

## Discussion

Dengue remains a significant global public health challenge with costs to both the infected individual and the struggling health systems of dengue-endemic countries. At present, treatment is limited to supportive care. A therapeutic that can attenuate disease and prevent progression to severe disease would represent a highly significant advance with enormous benefits for both patients and health systems. There is growing observational evidence from the critical care field to suggest that statins may have a beneficial role in a number of conditions, such as sepsis, acute lung injury and pneumonia. Statins have beneficial pleiotropic effects, including stabilizing and anti-inflammatory effects on the endothelium. As endothelial dysfunction is so important in dengue pathogenesis, stabilizing effects at this site may prove to be clinically beneficial. In addition, statins have an excellent safety profile and a low cost. In view of this, we are optimistic about the potential benefit of lovastatin in dengue.

## Trial status

We expect that patients will start being recruited to this trial in November 2012.

## Abbreviations

ALT: alanine transaminase; CK: creatine kinase; DSMB: data and safety monitoring board; FDA: US Food and Drug Administration; IFN-γ: interferon-gamma; IL-6: interleukin-6; IL-10: interleukin-10; MICB: MHC class I polypeptide-related sequence B; NS1: non-structural protein 1; PLCE1: phospholipase C epsilon 1; TNF-α: tumour necrosis factor-alpha.

## Competing interests

The authors declare that they have no competing interests.

## Authors’ contributions

JW, NVVC, TTH, RP, CS, BW and JF conceived of the study and participated in its design. JW developed the study protocol with NTT, LTHT, NVH, MW, LM and NTPD. All authors read and approved the final manuscript.
